# Combining Abdominoplasty and Breast Procedures Under Tumescent Local and Spinal Anesthesia: A Retrospective Study

**DOI:** 10.1007/s00266-025-05386-7

**Published:** 2025-10-23

**Authors:** Matilde Tettamanzi, Federico Ziani, Giovanni Arrica, Edoardo Filigheddu, Claudia Trignano, Corrado Rubino, Emilio Trignano

**Affiliations:** 1 Department of Surgical, Microsurgical and Medical Sciences, Plastic Surgery Unit, University of Sassari, Sassari, Italy; 2 Department of Biomedical Sciences, University of Sassari, Sassari, Italy

**Keywords:** Mommy makeover, Abdominoplasty, Mastopexy, Augmentation mammaplasty, Breast reduction, Local tumescent anesthesia, Spinal anesthesia, Body contouring

## Abstract

**Background:**

The “Mommy Makeover” is a combined plastic surgery procedure designed to address aesthetic concerns commonly experienced by women after pregnancy and breastfeeding. This procedure typically includes abdominoplasty and breast surgery. Traditionally, these surgeries are performed under general anesthesia, but the use of tumescent local anesthesia (TLA) combined with spinal anesthesia presents several advantages, including enhanced safety, effective pain management, and reduced postoperative complications.

**Methods:**

This study analyzes the outcomes of 62 patients who underwent Mommy Makeover surgery with TLA and spinal anesthesia from 2011 to 2023. The TLA solution was composed of 25 mL of 2% lidocaine, 8 mEq of sodium bicarbonate, and 1 mL of epinephrine (1 mg/mL) diluted in 1000 mL of 0.9% saline solution. Spinal anesthesia was administered at the L1–L2 intervertebral level using 15–18 mg of ropivacaine in a 4 mL solution.

**Results:**

The patient ages ranged from 25 to 74 years (mean age 47 years, standard deviation 10.236), and BMI ranged from 24 to 35 (mean BMI 29.5, standard deviation 2.379). A total of 62 abdominoplasties were performed, 61 were horizontal (5 of these included hernia repair), and 1 was vertical. Breast procedures included 10 augmentation mammaplasties, 5 mastopexies without implants, and 30 mastopexies with implants, and 17 reduction mammaplasties. Additionally, 22 liposuction procedures of the flanks were performed. Major complications occurred in 14.5% of patients, including 1 case of hemorrhage requiring reintervention, 5 hematomas, and 3 seromas. Minor complications included 6 cases of abdominal wound dehiscence and 4 cases of breast wound dehiscence.

**Conclusions:**

Tumescent local anesthesia combined with spinal anesthesia is a highly effective and safe method for performing combined abdominal and breast surgery in the context of a “Mommy Makeover.” This technique provides significant benefits, including precise pain management, minimal postoperative side effects, and enhanced patient and surgeon satisfaction.

**Level of Evidence IV:**

This journal requires that authors assign a level of evidence to each article. For a full description of these Evidence-Based Medicine ratings, please refer to the Table of Contents or the online Instructions to Authors www.springer.com/00266.

**Supplementary Information:**

The online version contains supplementary material available at 10.1007/s00266-025-05386-7.

## Introduction

The “Mommy Makeover” is a popular plastic surgery procedure that aims to restore the pre-pregnancy body contour of women by combining abdominoplasty and breast augmentation, breast reduction, or mastopexy. This comprehensive approach addresses common post-pregnancy concerns such as abdominal laxity, excess skin, and changes in breast shape and volume [1, 2]. The final goals are to enhance the aesthetic appearance of the abdomen and breasts while ensuring minimal scarring and optimal patient satisfaction. Abdominoplasty, also known as a tummy tuck, is a well-established procedure in aesthetic surgery, primarily performed to remove excess abdominal skin and fat, tighten the underlying muscles, and correct herniation of the abdominal walls [3]. Traditionally, abdominoplasty is performed under general anesthesia; however, recent advancements have shown the efficacy of tumescent local anesthesia (TLA) combined with spinal anesthesia [4]. This approach minimizes the need for general anesthesia, reducing the risks associated with respiratory and hemodynamic instability and enhancing postoperative recovery. Breast augmentation and breast lift are equally critical components of the “Mommy Makeover” [5, 6]. These procedures address the loss of breast volume and sagging, which are common after pregnancy and breastfeeding. Like abdominoplasty, these procedures have traditionally been performed under general anesthesia. However, the integration of TLA offers similar benefits in terms of safety, pain management, and postoperative recovery.

This study aims to elucidate the outcomes of combining abdominoplasty and breast augmentation/mastopexy under TLA and spinal anesthesia. By analyzing patient records from 2011 to 2023, we aim to demonstrate that this anesthesia technique is a viable and advantageous alternative to general anesthesia for “Mommy Makeover” procedures. The findings highlight the procedural safety, patient satisfaction, and aesthetic results achievable with this innovative approach.

## Materials and Methods

This retrospective study was conducted on 62 female patients who underwent primary abdominoplasty and breast augmentation, breast reduction, or mastopexy as part of a “Mommy Makeover” procedure between 2011 and 2023 (Table 1). All procedures were performed in accordance with the safety regulations and clinical practice guidelines set forth by the Italian Ministry of Health. Preoperative screening, perioperative monitoring, anesthetic protocols, and infection prevention measures adhered strictly to national standards for outpatient surgical safety. The inclusion criteria included women aged 25–74 years, who sought surgical intervention for postpartum body contouring, had a stable body weight for at least six months prior to surgery, and were in good general health. Exclusion criteria included patients with significant comorbidities, BMI>35, history of complications with local anesthetic techniques, history of laparocele, age>75 years old, Brugada syndrome, or any cardiologic syndrome that could complicate the procedure, anxiety-provoking conditions. Moreover, patients with preoperatively diagnosed, symptomatic, or large laparoceles (ventral hernias) were excluded. However, in several cases, small, asymptomatic fascial defects were discovered incidentally during abdominoplasty and were repaired intraoperatively without altering the planned surgical course. Patients were suggested to take folin, vitamin B12, and cyanocobalamin one month before the surgery to elevate hemoglobin and iron levels. All procedures were performed under tumescent local anesthesia combined with spinal anesthesia. The TLA solution was prepared using 25 mL of 2% lidocaine, 8 mEq of sodium bicarbonate, and 1 mL of epinephrine (1 mg/mL) diluted in 1000 mL of 0.9% saline solution [7]. This solution was infiltrated into the surgical area to provide local anesthesia, vasoconstriction, and hydrodissection. Spinal anesthesia was administered at the L1–L2 intervertebral level using 15–18 mg of ropivacaine in a 4 mL solution and a 27G pencil-point spinal needle. The spinal injection was performed by an anesthesiologist under sterile conditions, ensuring adequate anesthesia of the lower abdomen and breast areas. The surgical protocol began with spinal anesthesia administration, followed by patient positioning and the infiltration of the tumescent anesthetic solution. Once the sterile field was prepared, the procedure commenced with abdominoplasty, which lasted approximately 1 hour and 20 minutes. While the first surgical team completed the abdominal closure, a second team initiated the breast surgery. Preoperative preparation involves detailed drawings to guide incision placement and ensure symmetrical and harmonious results. Preoperative markings were made with the patient in a standing position to delineate the extent of skin excision and the new position of the umbilicus for the abdominoplasty, and the breast augmentation, mastopexy, or reduction incision lines were marked based on the chosen technique (periareolar or superomedial pedicled inverted T mastopexy or breast reduction). The areas for liposuction were also marked.

**Table 1:** Table of patients by procedure: shows the number of patients divided by type of abdominal procedure, breast procedure, and additional procedures.

Abdominal procedure	Breast procedure	Additional procedure	Number of Patients
Abdominoplasty	Mastopexy with implants	Hips liposuction	4
Abdominoplasty	Mastopexy with implants	None	24
Abdominoplasty	Breast augmentation	None	10
Abdominoplasty	Mastopexy	None	5
Abdominoplasty	Reductive mammaplasty	Hips liposuction	13
Abdominoplasty + hernioplasty	Mastopexy with implants	Hips liposuction	1
Abdominoplasty + hernioplasty	Mastopexy with implants	None	1
Abdominoplasty + hernioplasty	Reductive mammaplasty	Hips liposuction	3
Vertical abdominoplasty	Reductive mammaplasty	Hips liposuction	1

For abdominoplasty, a horizontal incision was made from the anterior superior iliac spine on one side to the other, just above the pubic area. The skin and subcutaneous fat were elevated off the abdominal wall up to the costal margins. Excess skin and fat were removed, and the rectus abdominis muscles were plicated for a firmer abdominal contour. The umbilicus was repositioned through a new opening in the abdominal skin flap [8]. Diastasis of the rectus abdominis muscle was corrected, and hernia repair was performed when needed. The incision was closed in layers using absorbable sutures for the deep tissues and non-absorbable sutures for the skin. Two drains are placed just below the inferior incision in the pubic area [9]. Breast surgeries were tailored to the individual’s needs: Reduction techniques used the Wise pattern or vertical incisions, employing the superomedial pedicle to maintain adequate vascularity [10]. Mastopexy involved skin tightening and reshaping to elevate the breast with or without implants, while augmentation employed implants in a subpectoral or subglandular plane to enhance volume and shape [11–14]. Flanks liposuction was incorporated to refine contours further in 22 patients. The access incisions for liposuction must be incorporated into the abdominoplasty incision to avoid additional scars and stitches. The wounds were covered with standard dressings or negative pressure wound therapy (NPWT) dressings [15]. The total surgical duration was limited to 2 h and 30 min to prevent the need for urinary catheterization and to ensure the effectiveness of the anesthetic (range 130–185 min, interquartile range 140–165 min, and 42% of procedures exceeding the 150-min threshold).

**Fig. 1:**
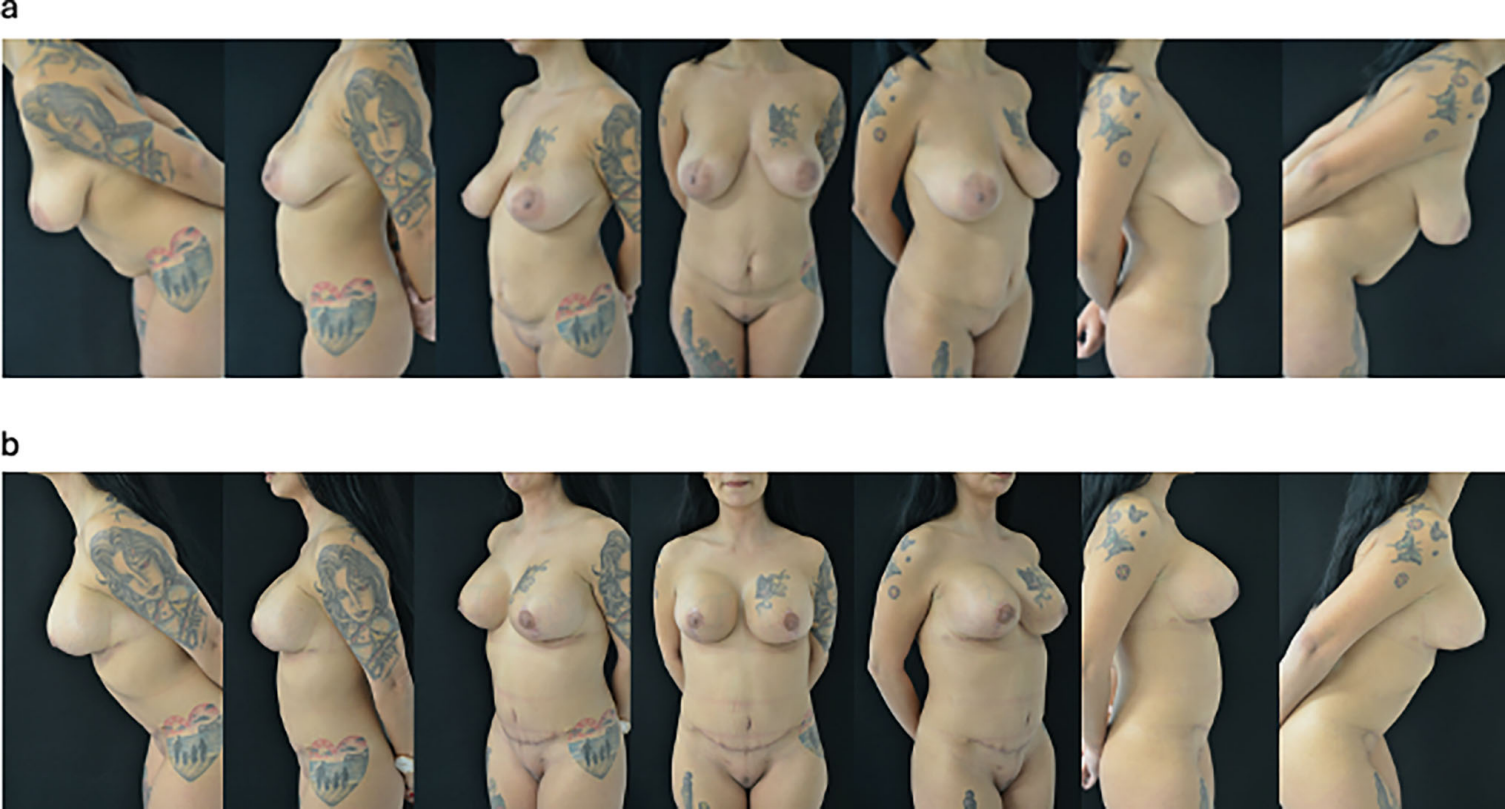
**a** Preoperative view of a patient undergoing abdominoplasty combined mastopexy with implants. **b** Postoperative view after 6 months.

**Fig. 2:**
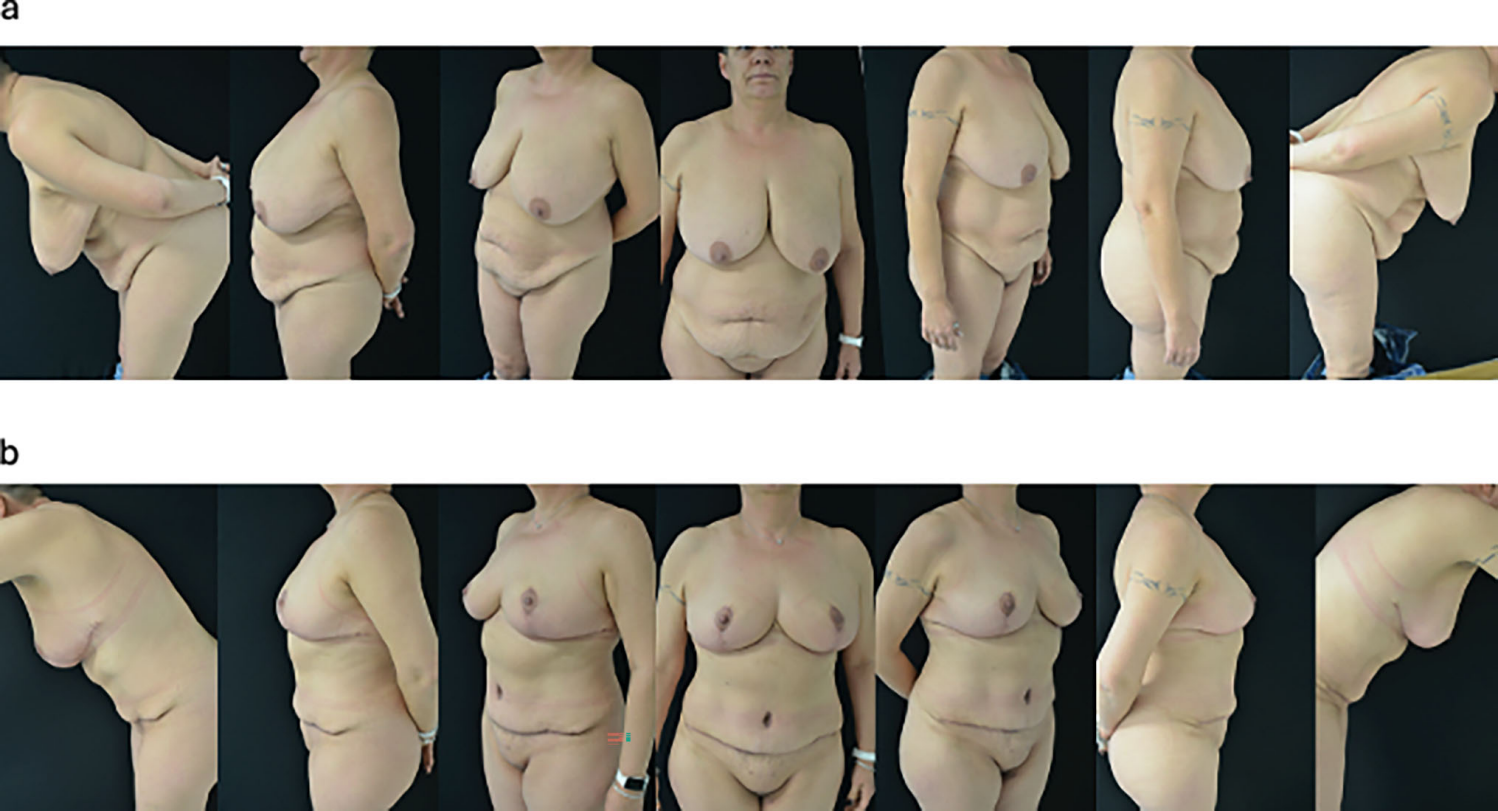
**a** Preoperative view of a patient undergoing abdominoplasty combined with breast reduction. **b** Postoperative view after 6 months.

Patients were monitored in the recovery room for an average of 240 min post-surgery. Standard postoperative care included pain management with oral analgesics, antibiotics to prevent infection, postoperative anticoagulant therapy (Seleparine), and detailed wound care instructions [16]. Patients were advised to wear compression garments for the abdomen and a surgical bra for breast support for six weeks postoperatively. Follow-up visits were scheduled at one week, one month, and three and six months post-surgery to monitor healing and address any complications (Figs. 1 and 2).

Primary outcome measures included intraoperative and postoperative complication rates [17], patient satisfaction with pain management and aesthetic results, and the duration of recovery. Complications such as hemorrhage, hematoma, seroma, infection, and the need for conversion to general anesthesia or reintervention were recorded. Patient satisfaction was assessed using a standardized questionnaire during follow-up visits [18, 19]. To assess patient satisfaction, we administered a standardized postoperative questionnaire designed to evaluate pain control, aesthetic satisfaction, and overall experience with the procedure (Table 2). The questionnaire was distributed during the six-month postoperative follow-up visit to ensure patients had sufficient recovery time to evaluate outcomes. It included both multiple-choice and 5-point Likert scale questions (ranging from “very dissatisfied” to “very satisfied”) covering domains such as pain during recovery, satisfaction with abdominal and breast aesthetic outcomes, willingness to repeat the procedure, and perceived improvements in quality of life. Responses were anonymized and evaluated, and descriptive statistics were used to report the satisfaction results. Due to the study’s retrospective nature and small sample size, no inferential statistical tests were applied to the questionnaire data, though this is identified as a limitation and will be addressed in future prospective studies.

**Table 2:** Raw response counts for patient satisfaction questionnaire (5-point Likert scale).

Question domain	Very dissatisfied	Dissatisfied	Neutral	Satisfied	Very satisfied
Pain control	0	0	0	4	58
Abdomen aesthetic	0	0	0	3	59
Breast aesthetic	0	0	0	4	58
Scar appearance	0	0	2	6	54
Belly button shape	0	0	4	6	52
Symmetry	0	0	1	9	52
Self-confidence	0	0	0	7	55
Overall satisfaction	0	0	0	9	53
Willingness to repeat	0	0	0	6	56
Willingness to recommend	0	0	2	3	57
Surgeon communication	0	0	3	3	56
Medical team professionalism	0	0	2	5	55

Total number = 62 patients.

## Results

Over a span of 12 years, this retrospective study analyzed the outcomes of 62 female patients who underwent combined abdominoplasty and breast augmentation, reduction, or mastopexy under tumescent local and spinal anesthesia. The patient ages ranged from 25 to 74 years, with a mean age of 47 years. The average duration of surgery was 150 min, with a mean recovery room time of 240 min. The surgery time was limited to a maximum of 2 h and 30 min to prevent the need for urinary catheterization and to ensure the effectiveness of the anesthetic.

Ten patients received bilateral breast augmentation, seventeen patients had a reduction mammaplasty, five patients underwent mastopexy without implants, and thirty patients mastopexy with implants. The mean volume of tumescent solution used per patient was 1150 mL, considering 450 mL (range 350–500 mL) for the abdomen, and 250–450 mL introduced per breast depending on the surgery, and no adverse effects related to lidocaine or epinephrine toxicity were observed. Notably, no cases required conversion to general anesthesia, demonstrating the effectiveness of the anesthesia protocol.

Postoperative complications were minimal, with a major complication rate of 14.5%, including 1 case of hemorrhage requiring reintervention, 5 hematomas, and 3 seromas (all in patients undergoing mastopexy with implants), which were managed conservatively without further surgical intervention (Table 3). Minor complications, observed in 16.1% of cases, included 6 cases of abdominal wound dehiscence and 4 cases of breast wound dehiscence (Table 4). Patient satisfaction was high, with 90% of patients reporting excellent pain management and aesthetic outcomes at the six-month follow-up. Pain and aesthetic satisfaction were assessed via separate items on the postoperative questionnaire, together with overall satisfaction, and willingness to undergo the procedure again. Most patients (95%) expressed a high level of satisfaction with their surgical results, particularly noting the natural appearance and enhanced contour of the abdomen and breasts. Specifically, patients were asked to rate their satisfaction with (1) postoperative pain control and (2) the aesthetic outcomes of both the abdomen and breasts, using independent 5-point Likert scales ranging from “very dissatisfied” to “very satisfied.” The 90% percentage refers to the proportion of patients who responded “very satisfied” to both the pain management and aesthetic outcome items, indicating concurrent satisfaction in these two domains. In contrast, the 95% value reflects patients who reported being “very satisfied” or “satisfied” with their overall surgical results, as measured by a broader item that asked about general satisfaction with the procedure. All patients were followed for at least one year, with no reports of long-term complications or dissatisfaction with the surgical outcomes.

**Table 3:** Major complications

Major complication	Number of patients
Hematoma (abdominoplasty)	5
Seroma (breast)	3
Hemorrhage (abdominoplasty)	1

**Table 4:** Minor complications

Minor complication	Number of patients
Abdominal wound dehiscence	6
Breast wound dehiscence	4

## Discussion

The combination of abdominoplasty and breast augmentation, reduction, or mastopexy in postpartum women, often termed a “Mommy Makeover,” has traditionally been performed under general anesthesia to ensure adequate analgesia and surgical conditions [20]. However, our study highlights the efficacy and safety of using tumescent local anesthesia combined with spinal anesthesia as a viable alternative to general anesthesia. This approach shows advantages in terms of minimizing intraoperative risks and improving postoperative recovery with comparable complication rates, which have been recurrent concerns in aesthetic surgery under general anesthesia.

General anesthesia is in fact widely used in body contouring procedures due to its ability to induce unconsciousness, ensuring that patients are entirely immobile and pain-free [21]. However, it is associated with a higher risk of respiratory depression, hemodynamic instability, and postoperative nausea and vomiting (PONV) [21, 22]. Studies such as that by Matarasso and Smith [1] and Gravante et al [23] report complication rates in combined breast and abdominal surgeries under general anesthesia to be as high as 15–20%, with complications ranging from deep vein thrombosis (DVT) and pulmonary embolism to more common issues such as wound infections, hematomas, and seromas. They emphasized that while combining abdominoplasty and breast surgery can enhance overall aesthetic outcomes, it may increase the complication rate compared to single procedure.

This increased risk is attributed to the prolonged operative time, larger surgical field, increased blood loss, and longer recovery phase associated with combined surgeries. Moreover, these procedures often require general anesthesia, which introduces additional risks such as postoperative nausea and vomiting, respiratory depression, and delayed recovery. For instance, Simon et al. [24] found that complication rates were significantly higher in combined surgeries exceeding 3 hours in duration, particularly when involving multiple anatomical regions. Cardoso de Castro et al. [25] similarly noted that seroma and hematoma formation were more frequent in combined procedures compared to isolated abdominoplasty. These findings underscore the importance of minimizing operative time, optimizing perioperative protocols, and carefully selecting candidates. In this context, our approach using tumescent local anesthesia and spinal anesthesia seeks to mitigate such risks by reducing operative stress, maintaining hemodynamic stability, and allowing early ambulation—thereby potentially lowering the incidence of thromboembolic and infectious complications often observed under general anesthesia.

Spinal anesthesia, while widely used and generally considered safe, is not without potential complications [26, 27]. Commonly reported adverse effects include post-dural puncture headache (PDPH), transient hypotension, urinary retention, and, in rare cases, transient or permanent neurological deficits. Other risks may involve nausea, bradycardia, or inadequate block requiring conversion to general anesthesia [28]. However, in our cohort, none of these complications were observed. All spinal blocks were administered by an experienced anesthesiologist using a consistent technique, which likely contributed to the absence of anesthesia-related adverse events. The safety of spinal anesthesia in our study aligns with previous findings suggesting that when properly performed in a controlled setting, it is a reliable alternative to general anesthesia for body contouring procedures.

Similarly to our work, Stevens et al. [29] demonstrated that combining aesthetic breast surgery with abdominoplasty should not have a statistically significant effect on morbidity. No statistically significant difference in complication rates was noted among the cohort of patients in Stevens et al study. Moreover, we were able to show that the use of TLA and spinal anesthesia mitigates these risks, providing a viable alternative to general anesthesia in combined surgeries. Notably, no patients in the current cohort required conversion to general anesthesia, and there were no reports of major anesthesia-related complications, such as respiratory depression or prolonged recovery times. These findings suggest that the combination of TLA and SA offers a safer intraoperative environment by maintaining spontaneous respiration and stable hemodynamics, which are benefits in lengthy combined procedures like the Mommy Makeover. The safety and efficacy of TLA in cosmetic surgery are well documented. The vasoconstrictive properties of epinephrine reduce intraoperative blood loss and help maintain clearer surgical planes, which is essential for procedures such as abdominoplasty that involve extensive tissue dissection [4, 30].

On one hand, spinal anesthesia, administered at the L1–L2 level using ropivacaine, offers targeted analgesia for both the abdominal and up to the sternal regions. On the other hand, ropivacaine is preferred due to its lower cardiotoxicity and longer duration of action compared to bupivacaine, making it ideal for prolonged procedures like the Mommy Makeover. Together, TLA and SA provide multilayered analgesia, reducing the need for supplemental intravenous analgesics or sedatives that are typically required in general anesthesia.

The study reports a major complication rate of 14.5%, which is comparable to the complication rates typically observed with general anesthesia. For instance, Pitanguy [31], Cardoso de Castro [25] and Simon [24] documented major complication rates ranging from 10 to 15% in combined breast and abdominal surgeries under general anesthesia. In our study, we were able to manage complications such as hematomas and seromas conservatively without requiring reoperation, reflecting the efficacy of the TLA and SA combination in minimizing intraoperative blood loss and postoperative fluid accumulation.

Additionally, minor complications such as wound dehiscence were observed in 16.1% of cases and were successfully treated via standard interventions. These minor complications fall within the lower range of what is commonly reported in the literature for Mommy Makeover surgeries under GA, where infection rates of 10–17% are typical [2, 32, 33].

One of the critical findings of this study is the high level of patient satisfaction with both pain management and aesthetic outcomes, with 100% of patients reporting excellent control of postoperative pain. This high satisfaction rate is consistent with reports in studies using local anesthetic techniques in plastic surgery [34, 35]. The avoidance of general anesthesia-related side effects, such as PONV and cognitive impairment, likely contributed to these favorable outcomes.

The average recovery time reported of 240 minutes observed in this study also aligns with the goal of enhanced recovery pathways in modern aesthetic surgery. Comparatively, surgeries under general anesthesia often necessitate longer recovery periods, with some patients requiring hospitalization due to nausea, vomiting, or other anesthesia-related complications. Moreover, early ambulation and discharge, facilitated by TLA and SA, reduce the risk of thromboembolic events, a major concern in surgeries under general anesthesia [36–38].

Several studies have explored the outcomes of using regional anesthesia techniques in combination with TLA for aesthetic surgeries. Our study group demonstrated the safety of using TLA with spinal anesthesia for abdominoplasty [4], showing a reduction in postoperative complications such as thromboembolic events and respiratory issues. The current study’s findings are consistent with these reports, further demonstrating a favorable recovery profile in this cohort of local and regional anesthesia as an alternative to general anesthesia [8, 9, 39]. General anesthesia often requires complex airway management, longer recovery times, and increased risk of postoperative nausea and vomiting (PONV). On the other hand, TLA, combined with spinal anesthesia, offers localized pain control and hemodynamic stability without compromising the depth of anesthesia required for major procedures like abdominoplasty. Moreover, tumescent anesthesia has the added benefit of vasoconstriction, reducing intraoperative bleeding and fluid loss, which can contribute to faster recovery and lower postoperative complications such as hematoma and seroma formation.

However, it is worth noting that the success of TLA and SA is highly dependent on surgeon and anesthesiologist expertise. Proper titration of local anesthetics and meticulous surgical technique are required to avoid complications such as local anesthetic systemic toxicity (LAST) and prolonged operative times, which could negate the benefits observed in this study.

This study has limitations inherent to its retrospective design. The relatively small sample size limits the statistical power and generalizability of the findings. Additionally, patient selection may have introduced bias, as only those deemed suitable for spinal and tumescent local anesthesia were included, potentially excluding higher-risk or more complex cases. The absence of a control group undergoing the same procedure under general anesthesia further limits our ability to draw direct comparative conclusions. Future prospective, randomized studies with larger cohorts are needed to validate these findings and further explore the benefits and risks of this anesthetic approach. Furthermore, we recognize the value of using validated patient-reported outcome measure such as the BODY-Q instruments in future studies to enhance the reliability and comparability of patient-reported outcomes.

## Conclusions

In conclusion, the study successfully demonstrates the safety and efficacy of combining tumescent local anesthesia with spinal anesthesia for “Mommy Makeover” procedures. Although no direct comparison with general anesthesia was undertaken in this study, this method appears to offer advantages in recovery profile and patient satisfaction within the study cohort, with comparable complication rates. Future studies may further investigate the long-term outcomes and broader applications of this anesthesia technique in other combined surgeries.

## Supplementary Information

Below is the link to the electronic supplementary material.

Supplementary file1 (DOCX 27 KB)
