# Supplementary material for: Combining Abdominoplasty and Breast Procedures Under Tumescent Local and Spinal Anesthesia: A Retrospective Study
Source: Aesthetic Plast Surg. 2025 Oct 23;50(11):4111–9. doi: 10.1007/s00266-025-05386-7 (PMC13315463; doi:10.1007/s00266-025-05386-7)
Supplement: Supplementary file 1 — Supplementary file1 (DOCX 27 KB) [file 266_2025_5386_MOESM1_ESM.docx]

Postoperative Patient Satisfaction Questionnaire

**Instructions:** Please answer the following questions based on your experience during and after your surgery.

Use the 5-point scale provided: 1 = Very Dissatisfied, 2 = Dissatisfied, 3 = Neutral, 4 = Satisfied, 5 = Very Satisfied.

## Section 1: Pain Management and Physical Recovery

1. How satisfied were you with the control of pain during the first 48 hours after surgery?
2. How satisfied were you with the comfort level during your recovery period (first two weeks)?
3. How satisfied were you with your ability to resume everyday activities within the first month?
4. How satisfied were you with the clarity of the pain management instructions provided?

## Section 2: Aesthetic Outcomes

1. How satisfied are you with the appearance of your abdomen after surgery?
2. How satisfied are you with the appearance of your breasts after surgery?
3. How satisfied are you with the symmetry of your breasts?
4. How satisfied are you with the position and shape of your belly button (umbilicus)?
5. How satisfied are you with the appearance of your surgical scars?

## Section 3: Overall Satisfaction and Psychological Impact

1. How satisfied are you with the overall results of your surgery?
2. How satisfied are you with the naturalness of your appearance and enhanced contours after surgery?
3. How satisfied are you with how your body looks in clothing?
4. How satisfied are you with how your body looks unclothed?
5. Has the surgery improved your self-confidence?

## Section 4: Willingness and Recommendation

1. Would you undergo the same procedure again, knowing what you know now? (1 = Definitely not, 5 = Definitely yes)
2. Would you recommend this procedure to a friend or relative? (1 = Definitely not, 5 = Definitely yes)

## Section 5: Satisfaction with Care

1. How satisfied were you with the communication and availability of your surgeon?
2. How satisfied were you with the professionalism and support of the medical team during your stay?
3. How satisfied were you with the quality of information you received preoperatively?
